# An examination of the Kuleshov effect using still photographs

**DOI:** 10.1371/journal.pone.0224623

**Published:** 2019-10-31

**Authors:** John Mullennix, Jeremy Barber, Trista Cory

**Affiliations:** University of Pittsburgh at Johnstown, Johnstown, PA, United States of America; University of Wuerzburg, GERMANY

## Abstract

The goal of the present study was to examine whether the effect of visual context on the interpretation of facial expression from an actor’s face could be produced using isolated photographic stills, instead of the typical dynamic film sequences used to demonstrate the effect. Two-photograph sequences consisting of a context photograph varying in pleasantness and a photograph of an actor’s neutral face were presented. Participants performed a liking rating task for the context photograph (to ensure attention to the stimulus) and they performed three tasks for the face stimulus: labeling the emotion portrayed by the actor, rating valence, and rating arousal. The results of the labeling data confirmed the existence of a visual context effect, with more faces labeled as “happy” after viewing pleasant context and more faces labeled “sad” or “fearful” after viewing unpleasant context. This effect was demonstrated when no explicit connection between the context stimulus and face stimulus was invoked, with the contextual information exerting its effect on labeling after being held in memory for at least 10 seconds. The results for ratings of valence and arousal were mixed. Overall, the results suggest that isolated photograph sequences produce a Kuleshov-type context effect on attributions of emotion to actors’ faces, replicating previous research conducted with dynamic film sequences.

## Introduction

Facial expressions are important cues to emotion [1, 2]. We rely much on facial expression to monitor and assess the mood or emotional state that another person is experiencing in a given moment. Although it is tempting to assume that facial expressions in isolation are sufficient to convey emotional state, there is much evidence suggesting that the context surrounding the viewing of a face affects the interpretation of facial expression [3–8]. Context consists of factors such as body posture, the social situation, cultural background, the visual scene, the presence of other faces, and verbal information [8,9]. The interpretation of facial expression appears modified by context, suggesting that the structural features of a human face alone may not properly convey emotion [9].

In the present study, the focus was on visual context and how such context affects the interpretation of facial expression. Research has shown that contextual information present in a visual scene is combined with facial expression and affects judgments about emotion [10–11]. In this research, the contextual information in the scene appears simultaneously with a target face. Research studies have also shown that visual context in the form of images presented in a dynamic flow that come before or after a facial stimulus affects interpretation of facial expression [12–15]. In this situation, the contextual images and the facial target image are presented closely together in time. This particular line of research is predicated on the classic Kuleshov Effect in film. First discovered by the Soviet filmmaker Lev Kuleshov in the early 1900s silent film era, the effect concerns contextual information in a film shot that affects the perception of an actor’s neutral face shown in a preceding film shot. The classic demonstration of the effect involved Kuleshov and his protégé Pudovkin showing film shot sequences to an audience [16]. There were three shot sequences consisting of a close-up shot of the actor Ivan Mosjukhin displaying a neutral expression followed by either a bowl of soup, a dead woman in a coffin, or a little girl playing. Pudovkin said that the audience viewing the sequences reported three different judgments of Mozhukin’s facial expression: heavy pensiveness, deep sorrow, and happiness, respectively. Thus, although the shot of the actor’s neutral face was identical in all three scenarios, the context provided by the subsequent film shot affected the audience’s interpretation of the actor’s emotion conveyed by his facial expression.

Over the years, there has been some controversy about this work [17]. As noted by Prince and Hensley [12], there are questions about the exact procedures used by Kuleshov and Pudovkin, the footage used, and varying accounts of their findings. Nevertheless, the Kuleshov effect has entrenched itself into the academic literature on film editing and the use of the montage. Over time, there have been attempts to empirically demonstrate the existence of the Kuleshov effect. Prince and Hensley constructed three film sequences similar to Kuleshov’s sequences, with the actor’s face shown before and after the contextual film shot of a woman lying in a coffin, a bowl of soup, or a child playing with a teddy bear. They showed the sequences to undergraduate students who were asked to identify the emotion that the actor’s face was portraying. They found that most of the respondents reported no emotion. They concluded that there was little evidence the Kuleshov effect exists [12]. However, their conclusions have been challenged by other research. For example, Wallbott [6] showed video clips from films and television dramas containing emotion-arousing situations followed by an actor’s face expressing various emotions. Wallbott found evidence that the attribution of emotion to an actor was significantly affected by the preceding video context [6].

More recently, the Kuleshov paradigm has been adopted by researchers examining the influence of visual context on interpretation of facial expression. Mobbs et al. [13] presented video sequences to viewers consisting of dynamic (zooming in or out) pictures rated for positive, neutral, or negative emotional valence followed by a face stimulus (neutral faces or faces morphed to create subtle happy or fearful expressions). They found that the dynamic context varying in valence that preceded the faces affected both behavioral and physiological measures related to the face stimuli. For behavioral measures, ratings of the faces related to certain dimensions of facial expression and mental state were affected by context. For physiological fMRI brain-related measures, context produced differences in brain activity located in the bilateral temporal pole, the STS insula, and the ACC. The results suggested that these brain regions are involved in the storage and coordination of contextual frames. Furthermore, they suggested that the observed activation of the vPFC region of the brain (an area related to the processing of top-down signals) in their study was consistent with the idea that top-down cognitive expectations based on activation of stored schemata were responsible for the effects of context.

Another study examining the effect of visual context on interpretation of facial expression was conducted by Barrett et al. [14]. Barrett et al. constructed films consisting of face-context-face sequences involving five different emotion contexts: happiness, sadness, hunger, desire, and fear. The context was composed of objects or events that the person in the film was looking at. Participants made three judgments of the actor’s face: emotional valence, arousal, and emotion category. Behavioral responses for emotion category indicated that the emotion related to the context for each of the five conditions produced more labeling of the actor’s face for that contextual emotion than the other choices. In addition, ratings of valence and arousal varied across contexts. The results suggested that the visual context affected viewers’ interpretation of facial expression. Barrett et al. [14], suggested that the effect could be due to one of three possible mechanisms: the observer directly perceiving emotion in the actor’s face, the observer inferring what the actor’s emotional state was using a form of cognitive inference, or the observer experiencing the actor’s emotion as a form of empathy. However, their results did not distinguish between these three possibilities.

Further evidence on the effects of visual context using a Kuleshov-style paradigm was obtained by Calbi et al. [15]. Calbi et al. used dynamic face-context-face film sequences where the effect of context (where context was a visual scene the person was looking at) was examined in terms of evaluations of valence, arousal, and emotion category for the actor’s face. They examined three visual scene context conditions: happiness, fear, and neutral. For behavioral responses, they found that interpretation of the actor’s face was affected by context, with faces labeled happy more often in the happiness condition and fearful more often in the fearful condition. In terms of valence, faces were rated as more positive in the happiness condition and more negative in the fear condition. For arousal, viewers rated faces as more arousing when preceded by a fearful context. Based on high amplitude Late Positive Potential (LPP) EEG data observed when faces were preceded by emotional context, Calbi et al. suggested that a cognitive process of attribution of expectations set by context accounted for how facial expression was interpreted, since the LPP is activated when evaluating incongruent sequences of stimuli. In other words, context sets up expectations and then those expectations are evaluated as congruent or incongruent with the facial stimulus when it is encountered.

The studies above show that sequential context presented before or after an actor’s face is factored into the viewer’s interpretation of the facial expression from the actor. It should be noted that in these studies the researchers used dynamic film sequences, either composed of photographs strung together and shown in rapid succession, or continuous film shots which included an actor’s face. Except for Mobbs et al. [13], who placed an interval of time between a context movie and the following face stimulus, other studies, such as Barrett et al. [14] and Calbi et al. [15], used sequences where the face shot appeared immediately after the context shot, which for all practical purposes is close to the simultaneous presentation of context used by Barrett and Kensinger [10] and Righart and de Gelder [11]. This brings up the issue of whether such context effects are time-sensitive and whether context can exert an effect when it is removed in time from labelling a facial stimulus. Although Mobbs et al. [13] observed context effects with some time elapsing between context and face, they also told participants that the following face shot was in response to the previous context movie.

In the present study, we examined whether a visual context effect on interpretation of facial expression could be produced by using still images unrelated to a facial stimulus where a substantial time gap between the context stimulus and the labeling of the actor’s facial emotion occurred. In this study, no instructions were provided to participants to explicitly connect the visual context to the following face. The reason these manipulations are important is that they provide a means to test the boundaries of the visual Kuleshov-style context effects observed by others. If the contextual framing effects observed by others dissipate when the context is not tied to the following facial stimulus, or if the effects dissipate when viewers are not exposed to dynamic context, this sets up important constraints on the effects of context on facial interpretation. If the effects of context are time-sensitive, this is also important. Any cognitive mechanism proposed to be involved in these context effects would have to account for these data.

To accomplish this goal, we manipulated the affective content (positive, negative, or neutral) of a context photograph that preceded an actor’s neutral face to examine whether the context affected the interpretation of emotion from the actor’s face when no explicit connection between the two photographs was presented. If a preceding contextual image presented upstream in time affects the labeling of emotion and/or ratings related to emotion and arousal for an actor’s face shown in a subsequent photograph, this would indicate that the visual context effect is robust and is not dependent on close timing between context and face. This would also suggest that information in memory about the preceding context produces the contextual effect.

One important issue regarding previous studies [6, 12–15] is stimuli. When examining these studies, there is considerable variation across them in terms of how stimuli were chosen and how they were presented in the experiment. For example, Prince and Hensley [12] filmed an actor and objects to create their film sequences, with no a priori assessment of the affective content of the contextual image or the neutrality of the actor’s face. Walbott [6] used video clips from films and television, with two judges determining the contextual content and emotional expression of the actors’ faces. Barrett et al. [14] used neutral faces from a standardized database, but the context images and videos were drawn from online sources without any objective a priori screening to determine affective content. Standardized neutral faces were also used by Mobbs et al. [13] and Calbi et al. [15]. Mobbs et al. also used context images pre-rated for emotional valence. In some studies, a context image was followed by a face [6,13]. In other studies, a face was presented followed by an image followed by the face again [12,14,15]. In the present study, careful attention was paid to the selection and screening of stimuli. The context photographs were pre-screened using objective ratings of pleasantness, with pleasant, unpleasant, and neutral photographs selected. This screening was performed to ensure some consistency among the contextual images in the affective content they contained. The neutral faces were selected from a standardized database of male and female faces. They were pre-screened for rated attractiveness and were pilot tested to ensure the facial expressions depicted by the actors were truly perceived as neutral. In terms of the photograph sequences, we were primarily interested in the effects of preceding context on faces, thus we used a series of two-photograph sequences where a context photograph was followed by a photograph of an actor’s neutral face. Although not exactly mirroring the face-context sequence purportedly used by Kuleshov [16], the sequence was similar to those used in previous empirical studies where the face followed context [6,12–15].

Another methodological issue present in previous studies was presentation time for the images contained in the film sequences. In the studies mentioned above [6,12–15], presentation times for the images presented within film sequences were controlled by the experimenter. Presentation times for images and faces across studies were highly variable, with times for context images ranging from 3 sec to 16.3 sec and presentation times for faces ranging from 750 ms to 7 sec. It is possible that the amount of time a viewer has available to examine context images and faces could impact the results, especially with brief presentation times that could increase the cognitive load on the viewer. In the present study, presentation time for the images was self-paced. Participants determined how long they viewed the contextual image and the face image on each trial before proceeding. This arrangement ensured that viewers had sufficient time to process the images before responding to them. Viewing times for the images were also recorded.

A third methodological issue was the absence of behavioral responses to context images in these studies [6, 12–15]. While the primary reason for this was to emulate the original Kuleshov demonstration, it is difficult to assess the degree to which participants attended to context images and processed the affective content contained in them when no response to the context images was required. In the present study, liking responses for the context images were required in addition to the labeling and rating responses for the facial stimuli. This ensured that all stimuli within the sequences were actively attended to.

The hypotheses for the present study were straightforward. If the affective content contained in context photographs presented prior to an actor’s face exerts an influence on the interpretation of facial expression, then we expected the labeling of emotion for the faces to deviate from the neutral expression depicted by the actor. We did not expect this deviation to occur on the majority of trials, as we expected the label “neutral” to still be selected most often. For pleasant context, we expected some faces to be labeled as “happy” more often compared to a neutral context. For unpleasant context, we expected some faces to be labeled as “sad,” “fearful,” or “disgusted” more often compared to a neutral context. For ratings of valence for faces (whether the actors were perceived as having “positive” or “negative” feelings), we expected that valence would be perceived as more positive for pleasant context and as more negative for unpleasant context, compared to a neutral context. For ratings of arousal for faces (whether the actors were perceived as “calm” or “excited”), we expected that the actors would be perceived as calmer for pleasant context and more excited for unpleasant context.

## Materials and methods

### Participants

Forty-four students (18 men and 26 women) enrolled in Introductory Psychology classes at the University of Pittsburgh at Johnstown participated. Average age was *M* = 19.5 (*SD* = 2.3), age range 18–32. All participants provided written informed consent prior to the study. Participants received course credit for their participation. Treatment of participants was in accordance with the ethical standards of the Declaration of Helsinki and the American Psychological Association ethical guidelines. The study was pre-approved by the University of Pittsburgh Institutional Review Board.

### Materials

Fifty-four context color photographs were drawn from the International Affective Picture System (IAPS) [18]. The IAPS photographs have ratings of pleasantness, arousal, and dominance. Three sets of 18 context photographs were selected: Neutral, Pleasant, and Unpleasant. The context photographs were selected via the IAPS Pleasantness ratings (1–9 scale ranging from unpleasant to pleasant). IAPS Arousal ratings (1–9 scale ranging from calm to excited) were also tabulated but not used for selection. The neutral photographs averaged 5.18 in pleasantness and 3.78 in arousal, the pleasant photographs averaged 7.92 in pleasantness and 4.23 in arousal, and the unpleasant photographs averaged 2.31 in pleasantness and 5.70 in arousal. T-tests indicated that both pleasantness and arousal ratings differed significantly across all three context photograph types. The pleasant stimuli consisted of photographs of animals, people, and nature. The unpleasant stimuli depicted people, acts or symbols of violence, and scenes/objects eliciting disgust reactions. The neutral stimuli depicted people and objects.

The neutral emotion face stimuli were drawn from the Karolinska Directed Emotional Faces (KDEF) database [19]. This database consists of color photographs of male and female actors, ages 20–30, exhibiting various facial expressions of emotion. Neutral emotion faces were selected with a straight-on angle (the actor looking straight into the camera). A straight-on angle was used to minimize the illusion that the actor was “looking” at the previous photograph. This was in contrast to previous studies where ¾ profiles or full profiles of actors’ faces were used to create smooth dynamic film sequences [14,15]. To ensure that the neutral faces were perceived as neutral, all emotional expressions from 35 male faces 35 female faces in the KDEF database were pilot tested with 18 participants who labeled each face with one of eight facial emotion labels (angry, fearful, disgusted, happy, sad, surprised, neutral, or indistinct). From these data, nine male neutral faces and nine female neutral faces were selected. For the selected male faces, an average of 15.9 out of 18 participants labeled the selected faces as “neutral.” For the selected female faces, an average of 15.2 out of 18 participants labeled the selected faces as “neutral.” In addition, ratings of attractiveness were obtained for the male and female faces on a 1–7 scale from “Very unattractive” to “Very attractive.” An attempt was made to choose faces that were neither extremely low or extremely high in attractiveness in order to represent “average” faces. The overall average attractiveness rating for the male faces was 2.97 (SD = 0.45) and the overall average attractiveness rating for the female faces was 3.55 (*SD* = 0.35).

### Procedure

Control of the experiment and data collection were performed using the E-Prime 2.0 software package [20]. The experiment was conducted on a laptop computer in a quiet laboratory room. Participants were run individually. The laptop screen was 15.6 inch with a screen resolution of 1366 x 768 pixels.

Fifty-four two-photograph sequences were presented, with 18 sequences per context condition. There were three context conditions: Neutral Context, Pleasant Context, and Unpleasant Context. For each sequence, a context photograph from the IAPS database was presented followed by a facial photograph from the KDEF database. Participants were told that they would see two different types of photographs on each trial in the experiment and that they were to make different responses to them. They were told they would see a real-life photograph presented first on each trial and rate it for how much they liked it. Then, they would see a photograph of a person’s face presented second on each trial and would judge the emotion that they believed the person was feeling based on their facial expression, the degree to which they thought the person was feeling positive or negative (in terms of mood), and how calm or excited they appeared. They were told that the real-life scenes were photographs shot by photojournalists and were not posed or fake. They were told that the photographs of people’s faces were taken when they were viewing stimuli that provoked different emotional reactions and that they would see different photographs taken of the same people throughout the experiment. No mention was made of any connection between the context photograph and the facial photograph presented in each sequence.

Participants pressed a key to initiate the two-photograph sequence. After the program presented the context photograph, participants viewed the context photograph for as long as they wished. When they were finished viewing it, they pressed a key to proceed to the next screen that presented a rating scale for liking. They rated the context photograph on a 1–7 scale from “Disliked the photograph” to “Liked the photograph.” This response was included only to ensure that participants attended to the context photograph. After they made their rating response, the program proceeded and then presented the facial photograph. Participants viewed the face for as long as they wished. When finished, they pressed a key to proceed to the first rating scale related to the face. The first scale was valence. For valence, they were told to rate the degree to which they thought the person in the photograph was experiencing a positive or negative feeling on a 1–9 scale from “Negative feeling” to “Positive feeling.” After making their response, the program proceeded to the arousal rating scale. They were told to indicate on a 1–9 scale (from “Calm” to “Excited”) the degree to which they thought the person in the photograph felt calm or excited. After they made their response, the program proceeded to the emotion labeling response screen. Participants were told to judge the emotion they thought the person was feeling based on their facial expression. They were presented with seven options: Angry, Fearful, Disgusted, Happy, Sad, Surprised, and Neutral. They were told to select the label that best matched the emotional expression of the person’s face and to press a key to indicate their response. After responding, the program proceeded to the next two-photograph sequence.

In each of the three conditions, nine male and nine female faces were presented. Each facial stimulus was presented three times in the experiment, once for each context condition, to control for any extraneous variables related to the faces (such as rated attractiveness, gender, etc.). The 54 sequences were randomized.

The experiment was self-paced. Participants were told they could take as much time or as little time as they wished to view each photograph before pressing a key to proceed to the subsequent response screen. For the context photographs, the image viewing time and the liking response were recorded. For the facial photographs, the viewing time and the valence, arousal, and labeling responses were recorded.

## Results and discussion

### Analysis of context photograph liking data

The liking ratings were analyzed via a one-way repeated measures ANOVA for the factor of context condition (neutral, pleasant or unpleasant). There was a significant main effect of context condition on liking rating, *F*(2,86) = 485.15, p < .001, η_p_^2^ = .92, with *M =* 6.25 for the pleasant condition, *M =* 4.24 for the neutral condition, and *M =* 1.77 for the unpleasant condition. These ratings verified that participants perceived the photographs across context conditions as differing in pleasantness.

Viewing time for context photographs was also analyzed via a one-way repeated measures ANOVA. No significant effect of condition on context photograph viewing time was obtained (*F =* .14), *M =* 2970 ms for the pleasant condition, *M* = 2929 ms for the neutral condition, and *M* = 3011 ms for the unpleasant condition.

### Analysis of facial emotion labels

The emotion category labeling data are shown in terms of the number of times a specific label was used (e.g., Angry, Fearful, Happy) to label the emotion of the actor’s neutral face. Given that there were 18 trials per context condition, the number of average times a label was used was out of a possible 18 trials (e.g., “angry” was used an average of 2.24 times per 18 faces in the neutral context condition). The labeling data are shown in Table 1. The most common response for face labeling was “Neutral,” which was expected. However, evidence for a Kuleshov-type effect lies with the use of labels other than “Neutral” across the context conditions. The emotion category labeling data was analyzed via a two-way ANOVA for context condition (neutral, pleasant, and unpleasant) and emotion category (Angry, Fearful, Disgusted, Happy, Sad, Surprised, and Neutral). There was no significant main effect of context condition (*F* = 2.2). However, there was a significant main effect of emotion category, *F*(6, 258) = 54.68, p < .001, η_p_^2^ = .56, and a significant interaction of context condition with emotion category, *F*(12,516) = 5.11, p < .001, η_p_^2^ = .11.

**Table 1:** Mean number of responses per emotional category (out of 18 possible responses per context condition) across neutral, pleasant, and unpleasant context conditions for face stimuli.

Emotion Category	Neutral Condition	Pleasant Condition	Unpleasant Condition
Angry	2.24 (*SD* = 1.74)	2.52 (*SD* = 1.95)	2.32 (*SD* = 1.85)
Fearful	1.48 (*SD* = 1.50)	1.45 (*SD* = 1.56)	1.91 (*SD* = 1.48)
Disgusted	1.48 (*SD* = 1.53)	1.93 (*SD* = 1.32)	2.00 (*SD* = 1.84)
Happy	1.86 (*SD* = 1.81)	2.30 (*SD* = 2.38)	1.14 (*SD* = 1.15)
Sad	2.50 (*SD* = 1.75)	2.68 (*SD* = 2.11)	3.55 (*SD* = 2.23)
Surprised	0.64 (*SD* = 0.87)	0.52 (*SD* = 0.93)	0.45 (*SD* = 0.70)
Neutral	7.84 (*SD* = 3.73)	6.59 (*SD* = 3.47)	6.57 (*SD* = 3.32)

A series of paired-samples t-tests were conducted to probe the interaction. The Holm-Bonferroni method was applied to correct for multiple comparisons. A summary of the results of the tests are shown in Table 2.

**Table 2:** Post-Hoc T-Test comparisons for the analysis of facial emotion labels.

Conditions Compared	t-value	df	p	significance
Angry/Neutral vs. Angry/Pleasant	-1.16	43	.25	n.s.
Angry/Neutral vs. Angry/Unpleasant	-.27	43	.79	n.s.
Angry/Pleasant vs. Angry/Unpleasant	.88	43	.38	n.s.
Fearful/Neutral vs. Fearful/Pleasant	.12	43	.91	n.s.
Fearful/Neutral vs. Fearful/Unpleasant	-1.72	43	.09	n.s.
Fearful/Pleasant vs. Fearful/Unpleasant	-2.23	43	.03	n.s.
Disgusted/Neutral vs. Disgusted/Pleasant	-2.17	43	.03	n.s.
Disgusted/Neutral vs. Disgusted/Unpleasant	-1.70	43	.10	n.s.
Disgusted/Pleasant vs. Disgusted/Unpleasant	-.25	43	.80	n.s.
Happy/Neutral vs. Happy/Pleasant	-1.63	43	.11	n.s.
Happy/Neutral vs. Happy/Unpleasant	2.98	43	.01	*
Happy/Pleasant vs. Happy/Unpleasant	3.40	43	.01	*
Sad/Neutral vs. Sad/Pleasant	-.62	43	.54	n.s.
Sad/Neutral vs. Sad/Unpleasant	-3.87	43	.01	*
Sad/Pleasant vs. Sad/Unpleasant	-2.45	43	.02	*
Surprised/Neutral vs. Surprised/Pleasant	.96	43	.34	n.s.
Surprised/Neutral vs. Surprised/Unpleasant	1.24	43	.22	n.s.
Surprised/Pleasant vs. Surprised/Unpleasant	.41	43	.68	n.s.
Neutral/Neutral vs. Neutral/Pleasant	3.10	43	.01	*
Neutral/Neutral vs. Neutral/Unpleasant	2.76	43	.01	*
Neutral/Pleasant vs. Neutral/Unpleasant	.07	43	.94	n.s.

* indicates significant at p < .05, two-tailed, after applying Holm-Bonferroni correction for multiple comparisons

The facial emotion labeling response data show evidence for a Kuleshov-type context effect. When the context was pleasant, more “happy” labels were used when judging the actors’ faces (compared to unpleasant context). When the context was unpleasant, more “fearful” labels were used (compared to pleasant context, although the comparison was not statistically significant), more “sad” labels were used (compared to neutral and unpleasant context), and less “happy” labels were used (compared to neutral and pleasant context). No differences across context conditions were shown for “angry” and “surprised,” which was expected given that the facial expressions for those emotions are far removed from a neutral facial expression. More “disgusted” labels were used when the context was pleasant and when the context was unpleasant (compared to neutral context), however these comparisons were not statistically significant.

An analysis of viewing time for the facial photographs was conducted via a one-way repeated measures ANOVA for the factor of context condition. There was a significant main effect of context condition on facial photograph viewing time, *F*(2,86) = 5.03, p < .01 η_p_^2^ = .10, with *M =* 3609 ms for the pleasant condition, *M =* 3505 ms for the neutral condition, and *M =* 3957 ms for the unpleasant condition. Post-hoc t-test comparisons showed that the viewing time for faces in the unpleasant condition was greater than both neutral and pleasant conditions, while the neutral and pleasant conditions did not differ.

### Analysis of facial valence

A one-way repeated measures ANOVA for the factor of context condition was used to analyze the rating data for facial valence. There was a significant main effect of context condition on facial valence rating, *F*(2,86) = 14.49, p < .001, η_p_^2^ = .25. The valence rating data are shown in Table 3. Posthoc t-tests showed that both neutral and pleasant conditions differed from the unpleasant condition, where the lower rating indicated that faces depicted in the unpleasant context condition were perceived as showing more negative emotion than in the other two conditions. Ratings across the neutral and pleasant conditions did not differ. This result suggests that the negative emotional content contained in the unpleasant context photographs affected perception of facial valence in the predicted direction.

**Table 3:** Ratings of valence and arousal across neutral, pleasant, and unpleasant context conditions for face stimuli (1–9 scales from “negative” to “positive” for valence and “calm” to “excited” for arousal).

	Neutral Condition	Pleasant Condition	Unpleasant Condition
Valence	3.58 (*SD* = 0.94)	3.50 (*SD* = 0.99)	3.10 (*SD* = 0.85)
Arousal	3.17 (*SD* = 1.08)	3.20 (*SD* = 1.10)	2.96 (*SD* = 1.05)

### Analysis of facial arousal

A one-way repeated measures ANOVA for the factor of context condition was used to analyze the rating data for facial arousal. There was a significant main effect of context condition on facial arousal ratings, *F*(2,86) = 4.90, p < .02, η_p_^2^ = .02. The arousal rating data are shown in Table 3. Posthoc t-tests showed that both neutral and pleasant conditions differed from the unpleasant condition, where the lower rating indicated that faces depicted in the unpleasant context condition were perceived as calmer than in the other two conditions. Ratings across the neutral and pleasant conditions did not differ. The finding of neutral faces being viewed as calmer after an unpleasant preceding photograph was unexpected. However, earlier it was mentioned that the arousal ratings for the unpleasant photographs were higher than the other conditions. It is possible that an arousal-based contrast effect was exhibited, where by a neutral face that follows a highly arousing unpleasant photograph is perceived as calmer (less arousing) than normal.

In terms of individual variability for the frequency with which participants used the label “neutral” for labeling the actors’ face, the average percent times “neutral” was used across context conditions was 38.9% (SD = 17.4%), varying from 19% to 94% across participants. Only six of 44 participants used “neutral” more than 50% of the time for their labeling responses.

## Conclusions

The emotion category labeling results provide evidence for a Kuleshov-type context effect using photographic stills consisting of highly controlled pre-screened stimuli. When labeling an emotion conveyed by an actor’s neutral face, the context effects observed were similar to the results observed by others using dynamic film sequences [13–15]. The use of “happy” labels increased for pleasant context and decreased for unpleasant context, and the use of “sad” labels increased for unpleasant context. These results fit our a priori predictions.

In terms of timing of events in this study, this was the sequence: Presentation of the context photograph, performance of the liking response, presentation of the face photograph, performance of the valence response, performance of the arousal response, and lastly performance of the emotion labeling response. The time to make the liking response to the context stimuli, the time to view the face photograph, and the time to perform the arousal and valence ratings were recorded. Across the three context conditions, the average time it took to proceed from initiating the liking response for the context photograph to performing the emotion labeling response for the face photograph was 10.2 sec. Thus, the information from the context photograph resided in memory for a substantial amount of time, with interceding tasks being performed. Yet, context effects on emotion labeling for faces were still obtained.

Viewing time for context photographs did not differ across context conditions. Viewing time for face photographs was longer for the unpleasant context condition compared to the other context conditions. It is difficult to pinpoint the reason for this latter result, but it is possible that additional viewing time was used to process several suitable labeling response options (neutral, sad, fearful, disgusted) invoked by unpleasant context, compared to only two labeling response options (neutral, happy) for pleasant context.

Context also affected ratings of emotional valence for faces. For valence, the results were mixed, in terms of our original predictions. Unpleasant context produced higher “negative” feeling ratings attributed to actors’ faces. However, pleasant context had no effect on valence, contrary to our prediction that higher “positive” ratings would be present. This result differed from Calbi et al. [15], who observed that both “fear” and “happy” contexts produced higher negative feelings and lower negative feelings attributed to neutral faces, respectively. However, our “pleasant” and “unpleasant” contexts do not exactly map on to their “fear” and “happy” contexts, so the comparisons are not exact.

For arousal, the results did not support predictions. Pleasant context had no effect on perception of arousal for actors’ faces, while unpleasant context resulted in higher “calm” ratings. Calbi et al. [15] found that their “happy” context had little effect on ratings of arousal faces, but the “fear” context produced higher ratings of arousal. For unpleasant context, we found the opposite effect. One possible explanation for this result is a contrast effect. The arousal ratings for unpleasant photographs were high compared to the arousal ratings for pleasant and neutral photographs. After viewing an unpleasant photograph that elicits strong arousal, perhaps when a neutral face follows the face is perceived as calmer than usual as the arousal level in the viewer lessens. With low-arousal context photographs in the pleasant condition, a contrast mechanism is not engaged. This is purely a speculative observation. It is also important to note that the effect size for arousal was small, thus we did not place much importance on it.

The most important results from the present study were the emotion category labeling data. The results indicated that a context effect on interpretation of facial expression can be elicited using isolated photographs shown in a two-photograph sequence where the context precedes the performance of a labeling response for the actor’s face by a substantial amount of time. This finding expands upon previous research using concatenated images strung together in dynamic film sequences [13–15], where the viewer perceives the actor’s face as reacting to the contextual film shot. Our finding is important because it demonstrates that context effects on facial expression do not have to be explicitly connected to a visual scene involving presentation of an actor’s face and the context does not have to be presented in dynamic film-related format to produce an effect. In addition, the information from the context persists in memory for at least 10.2 sec before affecting the subsequent labeling of facial emotion. This indicates that the context information is stored in the form of cognitive expectations or persistent affective information for a substantial amount of time. This suggests that the cognitive mechanism responsible for the context effects is memory-intensive, with the consequent need for attentional resources to hold the information in memory before labeling occurs.

Another important point regarding the present study was that the affective information in the context photographs was highly controlled and manipulated. This shows that the Kuleshov-style context effects observed by others using a variety of stimuli and presentation schemes are robust and generalize to stimuli specifically selected to vary in affect.

One issue in the present study was that we did not explicitly ascertain whether viewers may have believed there was a connection between the two photographs in the sequences used, as we used no post-test questions to assess this. Instructions to participants mentioned no connection between the photographs. The instructions simply explained the separate tasks to be performed for each photograph in the sequence. We believe it most likely that performing a liking rating task for the contextual photograph and performing three different rating tasks for the face photograph led viewers to believe that there was little to no connection between the photographs in the sequence. On the other hand, it is possible that viewers believed that the context photograph acted as a “prime” of some sort for face photograph on each trial. In the latter case, they may have believed that the two photographs were connected.

The data on valence and arousal ratings did not exactly fit a priori predictions. As explained above, there may be reasons for the pattern of results we observed for those measures. It would be worthwhile to further examine the absence of a pleasant context effect on valence, although other research examining context effects on perception of art has found that preceding pleasant context has weaker effects than unpleasant context on decisions for subsequent stimuli [21]. For the arousal finding, in future work it would be worthwhile to examine whether the finding that a face was perceived as “calmer” after presentation of a high-arousal context photograph is a robust finding able to be replicated.

Although some of the contextual images and face stimuli used in previous studies were pre-screened [6,12–15], the criteria used to select stimuli used across these studies varied substantially. One must always consider the possibility that artifacts can arise from stimuli that are not properly selected or screened. In the present study, we used context photographs and face photographs that were carefully selected and pre-screened. The context photographs were selected using objective ratings of pleasantness from the IAPS database and the face photographs from the KDEF database were pre-screened in a pilot study to ensure that the actors’ expressions were perceived as neutral. In addition, ratings of attractiveness for faces were also obtained, with the ratings used to select faces of “average” attractiveness so that low or high attractiveness would not be a possible unforeseen confounding factor. Altogether, we were confident our stimuli were suitable for the goals we outlined for the study.

Another difference between the present study and previous studies was that, in the present study, participants controlled the amount of time they spent viewing photographs to ensure they processed them sufficiently before making their responses. Participants also rated the context photographs for liking, thus ensuring that viewers paid active attention to the contextual stimuli. In previous studies, responses were only required for the face stimuli. In future studies, it would be interesting to manipulate the presentation time of images in stepwise fashion to assess what impact that has on the observed context effects. Comparing fast presentation times to slow presentation times may allow some insight into whether these context effects are due to System 1 “automatic” processing or System 2 “controlled” processing in the cognitive system [22].

Given that a Kuleshov-type context effect was obtained using highly controlled stimuli under conditions where attention to the stimuli was ensured, one may consider the nature of the psychological processes responsible for the effect. The present study was not designed to explicitly test the suggestions from previous research [13,15] that cognitive expectations produced by context are responsible for the Kuleshov-style contextual framing effect. However, our results are certainly consistent with the idea. It is likely that the perceived pleasantness of a preceding contextual photograph engages a host of stored cognitive and affective schemata that are activated upon viewing the photograph. Then, when a subsequent neutral face photograph is presented, the judgment of the emotion attributed to the actor’s facial expression is affected by the activated schemata. The results from our emotion labeling data clearly indicate that labeling is “moved” by the preceding context, showing that isolated photographic stills (where the dynamic flow between images is minimized compared to film sequences) produce a Kuleshov-type effect, just as concatenated film sequences do.

Finally, some may claim that the observed context effects are simply a form of general demand characteristic, where the labeling of the faces is determined by the viewer’s belief that the emotional category label for the face should match the affective content of the preceding context photograph. Taken to the extreme, this idea would require that the labels attached to faces should match the context 100% of the time. Obviously this did not occur. In terms of the original Kuleshov effect in film, whether the observed effects are due to demand characteristics or not may be irrelevant. If the film viewer believes that a film shot was designed by a director to produce a perception of emotion in the actor’s face, does that matter or does that affect the viewing experience? Some would say it would not.

In future work, studies should be designed to test further the cognitive and affective bases for the Kuleshov effect. As well, studies should examine how far the limits of contextual Kuleshov-type effects extend, in terms of image sequences. The results from such studies will be informative in terms of context effects in general and how they affection perception and cognition.
